# A new species of the gamasid mite genus *Arctoseius* Thor, 1930 (Parasitiformes, Mesostigmata, Ascidae) from Russia with a key to the *multidentatus* species-group

**DOI:** 10.3897/zookeys.313.5317

**Published:** 2013-06-28

**Authors:** Olga L. Makarova, Evert E. Lindquist

**Affiliations:** 1 Severtsov Institute of Ecology and Evolution, Russian Academy of Sciences, Moscow 119071, Russia; 2 Canadian National Collection of Insects, Arachnids and Nematodes, Science & Technology Branch, Agriculture & Agri-Food Canada, Ottawa, Ontario K1A 0C6, Canada

**Keywords:** Gamasid mites, Arctic, *Arctoseius koltschaki* sp. n., *multidentatus* species-group, identification key

## Abstract

A new gamasid mite species belonging to the genus *Arctoseius* Thor, 1930 is described from Russia. *Arctoseius koltschaki* **sp. n.** is distributed in the plain and mountain tundras from Khibiny Mountains to Chukotka on the north and to West Sayan Mountains on the south. A diagnosis and a key for identification of species comprising the *multidentatus* species-group (*Arctoseius multidentatus* Evans, 1955; *Arctoseius wisniewskii* Gwiazdowicz & Kamczyc, 2009; *Arctoseius sexsetus* Lindquist & Makarova, 2011; *Arctoseius haarlovi* Lindquist & Makarova, 2011; and *Arctoseius koltschaki* **sp. n.**) are given.

## Introduction

Arctic landscapes are populated by at least 26 mite species belonging to the genus *Arctoseius* Thor, 1930, which constitutes about half of the total diversity of this genus (Makarova in press). Such species richness appears to be unique among terrestrial mite genera within the Arctic. In spite of intensive investigations in the two last decades (Petrova and Makarova 1991; Makarova and Petrova 1992; Makarova 2000a, b; Lindquist and Makarova 2011), several arctic species still remain undescribed, one of which is treated here. It belongs to the *multidentatus* species-group, for which a diagnosis and a key to species are presented.

## Material and methods

In total 59 females, 23 males and 19 nymphs were found in 25 localities. Setal notation for the idiosoma follows Lindquist and Evans 1965, with some modification (Lindquist 1994). The leg and palpal chaetotaxy corresponds to Evans 1963, 1964. The poroidotaxy and adenotaxy are given according to Johnston and Moraza 1991, with small modification (Makarova 2003). The length of all shields was measured on the mid-line, the width at the broadest part, except for the sternal/sternitigenital shield, whose length was measured from the level of setae *st*1 to the posterior margin, and the width at the broadest part between levels of setae *st*2 and *st*3 (Makarova 2000a). The length of the epigynal shield was measured to include the anterior flap, the width – next to the posterior margin. The length of legs and tarsi are given excluding the ambulacrum, and also the pedicel on leg I. The subcapitulum length was measured from its anterior margin without appendages (internal malae, corniculi). The length of the chelicera excludes the basal segment, and the cheliceral digit length is based on the movable digit. Measurements were carried out in 20 females and 10 males and their limits are stated in micrometers (μm). The following ratios were used in the description (Makarova 2000a):

***lD*/*wD*** length-to-width ratio of the dorsal shield;

***J*(1–4)*min/jmax*** ratio of the length of dorsocentral setae inserted on the opisthonotal and podonotal regions, notably the shortest among *J*1–4 and the longest in the series *j*;

**(*J*4–*J*4)/(*J*3–*J*3)** ratio of transverse distances between bases of opisthonotal setae *J*4 and *J*3 in pairs;

***Z*5/(*Z*5–*Z*5)** ratio of setae *Z*5 length to the distance between them;

***lSt*/*wSt*, *lAn*/*wAn*, *lVA*/*wVA*** length-to-width ratios of the sternal, anal, and ventrianal (in males) shields, respectively;

***lCo*/*lD*** ratio of the length of the corniculus and of the dorsal shield, %;

***lCh*/*lD*** ratio of the length of the chela and of the dorsal shield, %;

***lCh*/*lCo*** ratio of the length of the chela and of the corniculus;

***lExI*/*lD*, *lT*IV/*lD*** ratio of the length of leg I or tarsus IV and of the dorsal shield;

***lT*III/*wT*III** length-to-width ratio of tarsus III, width taken at basistarsus.

In the male description, the features common with the female are omitted.

Holotype and most of paratypes are deposited in Zoological Institute, Russian Academy of Sciences, Saint Petersburg (ZIRAS). Part of paratypes, 5 females, 1 male, 2 N2, from Suntar-Khayata Range, 1500 m a.s.l. (above sea level), spotty tundra (on one slide) – in Canadian National Collection of Insects, Arachnids and Nematodes, Ottawa (CNC).

## Species description

### Arctoseius koltschaki

Makarova & Lindquist sp. n.

urn:lsid:zoobank.org:act:25C72458-8447-4610-BCA1-EC2EBC33D2A6

http://species-id.net/wiki/Arctoseius_koltschaki

**Figures 1–4.**
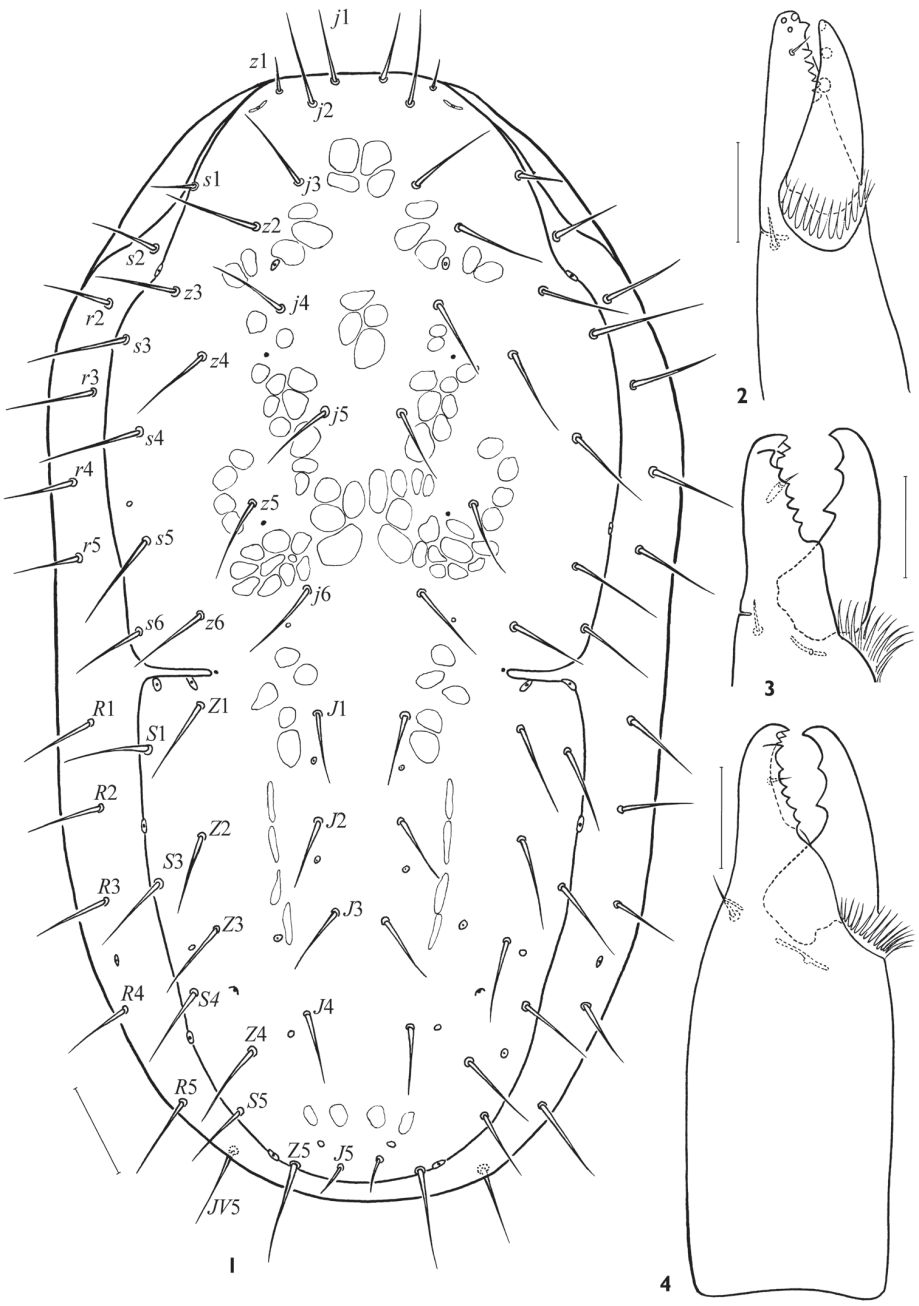
*Arctoseius koltschaki* sp. n., female **1** idiosomal dorsum **2–4** cheliceral digits. Scales: **1** – 50 μm, **2–4** – 25 μm.

Figs 1 [Fig F2] [Fig F3] [Fig F4] [Fig F5] –25

#### Material.

Holotype, female: EAST SIBERIA, YAKUTIYA, Khalerchinskaya Tundra, Chukochiya River, mosses in shrub tundra, IX 1975, leg. E.V. Gordeyeva (ZIRAS).

Paratypes: 5 females, 1 male, 2 N2, EAST SIBERIA, Suntar-Khayata Range, upper reaches of Kyubyume River, 63°13'N, 139°36'E, 1500 m a.s.l., spotty tundra, 29.VII 2002, leg. O.L. Makarova (CNC); 1 male, same district and collector, 1960 m a.s.l., snow bed near small stream, sedge turf, 1.VIII 2002; 1 female, delta of Indigirka River, Russko-Ustinskaya Protoka, hummocky *Dryas*-forb tundra, 14–16.VII 1994, leg. A.B. Babenko; NOVAYA ZEMLYA ARCHIPELAGO, 1 male, Southern Island, Pan’kova Zemlya, lower part of slope, sedge-willow-moss tundra, VIII 1995, leg. S.V. Goryachkin; 1 female, 1 male, Northern Island, Krestovaya Bay, tundra, VIII 1993, leg. V.I. Bulavintsev; 1 female, BOL’SHEZEMEL’SKAYA TUNDRA, Vorkuta Region, Sivaya Maska vicinity, tundra, 20.VII 1978, leg. N.A. Kuznetsova; 3 females, 1 male, KOLA PENINSULA, Khibiny Mountains, Eastern Petrelius Pass, *Dryas*-sedge-moss tundra, 28.VI 2006, leg. A.B. Babenko (ZIRAS).

Other material: NORTH-EAST ASIA, 5 females, 1 male, Chukotka, Daurkin Peninsula, lower reaches of Chegitun River, forb-sedge-moss tundra, 30.VIII 1996, leg. M.V. Berezin; 5 females, 3 males, Magadan Region, upper reaches of Kolyma River, Peak Aborigen vicinity, *Pinus pumila* litter, 24.VIII 2006, leg. A.V. Alfimov; 1 female, Olskoye Plateau, upper reaches of Ola River, 60°39'N, 151°16'E, 1220 m a.s.l., forb meadow, 11.VIII 2011, O.L. Makarova; EAST SIBERIA, delta of Kolyma River, Pokhodskaya Edoma, 1 female, litter in lower part of slope; 1 female, wet grassy meadow, 18.VII 1994, leg. A.B. Babenko; delta of Yana River, Shirokostan Peninsula, vicinity of Ledyanoye Lake, 72°25'N, 141°00'E, 1 female, south slope of valley, forb-grassy meadow; 1 male, high river terrace, *Dryas*-*Carex* tundra, 4–6.VIII 1994, leg. A.B. Babenko; 3 females, 1 N1, Yana Bay, Makar Island, 6.VIII 1985, leg. V.I. Bulavintsev; Suntar-Khayata Range, upper reaches of Kyubyume River, 3 males, 1 N2, 1300 m a.s.l., lichen *Larix*-forest, litter, 25.VII 2002, 2 females, 1 male, 1 N1, 1800 m a.s.l., snow bed, under *Rhododendron aureum*, 2 males, 5 N2, 2 N1, 1800 m a.s.l., hummocky *Eriophorum*-community, 28.VII 2002, 1 female, 1 N2, 1800 m a.s.l., lichen-willow tundra, 9.VII 2002, 1 female, 1 N2, 1 N1, 2000 m a.s.l., mossy tundra, 15.VII 2002, 4 females, 3 males, 2 N2, 1 N1, *Dryas*-*Carex*-*Eriophorum* bog, 19.VII 2002, leg. O.L. Makarova; 1 female, Cherskogo Range, Ust-Nera vicinity, 500 m a.s.l., grassy meadow near stream, 22.VII 1992, leg. N.A. Kuznetsova; TAYMYR PENINSULA, 1 male, N Taymyr, Khariton Laptev Coast, Opalovaya River, seaside sand-rubble terrace with *Salix polaris*, 15–17.VIII 1994, leg. A.B. Babenko; Taymyr Lake, Cape Blizhnyi, 2 females, *Tetraplodon* moss cushion in tundra, 28.VII 1994, 1 female, lemming hill within stony tundra on elevation, grassy turf, 10.VIII 1994, 2 females, lemming burrow, 17.VIII 1994, 5 females, 1 N1, lemming hill within spotty tundra, grassy turf, 17.VIII 1994, leg. O.L. Makarova; 1 female, NW Taymyr, upper reaches of Kolomejtseva River, lemming hill in spotty tundra, grassy turf, 21.VIII 1997, leg. O.L. Makarova; 2 females, 1 male, NW Taymyr, Ragozinka River, spotty tundra, 9.VII 1986, leg. A.B. Babenko; 1 female, NW Taymyr, mouth of the Tareya River, 73°15'N, 90°35'E, dry *Dryas*-community, 22.VII 2010, O.L. Makarova; 1 female, SW Taymyr, vicinity of Pyasino Lake, Nyapan’ Upland, zoogenic meadow on a hill, 16.VII 1999, O.L. Makarova; VAIGATCH ISLAND, 1 male, no other data, VII 1984, leg. V.I. Bulavintsev; NOVAYA ZEMLYA ARCHIPELAGO, 1 male, no other data, leg. V.I. Bulavintsev; BOL’SHEZEMEL’SKAYA TUNDRA, 3 females, Yugor Peninsula, Cape Belyi Nos, 25.VI 1983, leg. V.I. Bulavintsev; 1 female, Vorkuta city vicinity, IX 2009, leg. E.M. Perminova; 1 female, Pechora Bay, Kuznetskoye Lake, polar fox hill, litter under *Cornus suecica*, 25.VIII 1994, leg. A.B. Babenko; KOLGUEV ISLAND, 3 females, 1 male, 2 N2, 1 N1, tundra litter, VII 2011, leg. S.B. Rosenfeld; SOUTH SIBERIA, WEST SAYAN MOUNTAINS, 1 female, 50°23'N, 90°26'E, Tsagan-Shibetu Range, Mugur-Aksy vicinity, 2800 m a.s.l., tundra, litter, 22.VII 1993, leg. S.K. Stebaeva.

#### Description.

Adults of middle size, yellowish or brownish, with idiosoma rather narrow and appendages of normal proportions. Idiosomal shields moderately sclerotized, very finely punctate (punctation rarely visible), with clearly reticulate ornamentation only on sternal, genital or sternitigenital shields; dorsal shield smooth with distinct sigillae. Most body setae of moderate size, needle-shaped. Some setae of distal leg segments, especially of leg IV, elongated and finely tipped, subapical setae *av*-1 and *pv*-1 on tarsi II-IV strongly formed and blunt.

***Female*.** *Idiosomal dorsum*. Dorsal shield 528-616 × 248-304, narrowing posteriad, *lD*/*wD* ca 1.96–2.24, its maximal width at level of setae *s*4 (Fig. 1); lateral incisions of moderate length (30–44). Podonotal region normally with 17 pairs of simple setae (*z*3 present); *z*1 and *s*1 seldom asymmetrically absent; *s*2 usually on soft cuticle, sometimes asymmetrically on shield margin, sometimes symmetrically absent; *s*6 rarely off shield. Opisthonotal region with 13–14 pairs of setae (*S*2 always absent, *S*3 or *S*5 rarely asymmetrically off shield margin). Among podonotal setae, *z*1 (13–22) and *s*1 (20–35) distinguished by clearly shorter lengths, seta *j*1 30–36, *j*2 32–42, length of other setae 30–50. On opisthonotal region, setae *J*1-4 (25–35) slightly shorter than others (30–44), except *J*5 clearly shortest (9–17) and *Z*5 clearly longest (48–63); *J*(1–4)*min/jmax* 0.65–0.80, *Z*5/(*Z*5-*Z*5) 0.76–1.26; (*J*4-*J*4)/(*J*3-*J*3) 1.31–2.00. Dorsal shield with 5 pairs of gland pores: *gdj*4, *gdj*6, *gdz*5, *gdz*6, all poorly visible and *gdZ*3 distinct. All marginal setae on soft cuticle (their length 28–44, only *r*3 39–48); 4 (rarely 3) setae in series *r*, 5 setae in series *R*; marginal poroid *Rp* in usual position between setae *R*3 and *R*4.

*Idiosomal venter*. Base of tritosternum narrow (20–35 × 16–22); laciniae with sparse large barbs, free for ca 0.8–0.9 of lengths, their fused basal area fimbriated anteriorly (Fig. 20); rarely barbs are hardly separated (Fig. 21); length of laciniae free part 60–80. Presternal platelets large, consolidated with each other and sternal shield, lineate and clearly punctate (Fig. 6). Sternal shield commonly longer (146–164) than wide (124–160), *lSt*/*wSt* 1.00–1.23, merged with endopodal platelets between coxae II-III. Endopodal projections between coxae I-II small, nearly bacilliform, commonly separated, their lateral part encompassing opening of gland *gvb*. Sternal shield entirely reticulated; anterior margin straight or slightly concave, posterior margin straight. Sternal shield with typical setae *st*1-3 (32–44), lyrifissures *iv*1-3, and with vestiges of gland *gv*1 on posterior margin. Setae *st*4 (24–34) on soft cuticle. Endopodal strips between coxae III and IV free, rather narrow, partly hidden under epigynal flap. Epigynal shield (140–164 × 72–82) distinctly reticulated (Fig. 9), broadly axe- or flask-shaped, with evenly convex hyaline flap well distant from sternal shield, and posterior margin broadly convex; lateral margins widening behind level of setae *st*5, but *st*5 (26–36) and paragenital poroids *iv*5 remain on soft cuticle. Opening of gland *gv*2 on soft cuticle close beside end of peritrematal-exopodal strip behind coxa IV. Two pairs of postgenital platelets in fold of soft cuticle adjacent to posterior margin of epigynal shield, another pair between setae *JV*1 and *ZV*1. Anal shield rather small, ovate, weakly reticulated (Fig. 10), longer (94–112) than wide (76–93), *lAn*/*wAn* 1.08–1.30; paranal setae (36–46) variably inserted, usually between mid-level of anus and its posterior margin, and often nearly as long as postanal seta (42–64); opening of gland *gv*3 inconspicuous; cribrum ordinary. Opisthogastral setae (8 pairs, *JV*1–5, *ZV*1, 2, 4) of moderate length (most setae 30–40), *JV*5 longest (35–52), *ZV*1 shortest (20–34). Anterior metapodal platelets small, of variable form; posterior metapodal platelets elongate, often bacilliform (24–39 × 4–7). Exopodal platelets between coxae II and III, as well as between coxae III and IV, small, triangular, rarely visible in ventral aspect. Peritrematal shield reduced, of uneven width, with angular extension at level of coxa I; its anterior end abutting dorsal shield; its posterior edge connecting with exopodal strip enveloping coxa IV posteriorly; lyrifissures *ip*1–3 and glands *gp*1, 2 present. Peritreme shortened (128–156 × 8–11), extending to mid-level of coxa II anteriorly. Spermathecal apparatus seldom visible, with very narrow tubuli, thin-walled sperm receptacle and sperm duct strongly expanded medially (Fig. 8).

*Gnathosoma*. Gnathotectum (28–39 × 40–56) triramous, with projections of almost equal length, sometimes divided apically (Figs 23, 24); middle process sometimes narrower basally than lateral ones. Subcapitulum always slightly longer (116–128) than wide (104–120) (Fig. 7). Deutosternum with 7 (rarely 8) rows of denticles (15–22 denticles in each row); groove delineated laterally, its width 15–19 (Fig. 7). Hypostomatic pair *hp*3 (46–58) longer than other subcapitular setae (*hp*1 31–40, *sc*26–42, *hp*2 18–26); all setae simple, attenuate. Corniculi somewhat elongated, 46–53 × 15–19; *lCo*/*lD* 7.66–9.56. Internal malae slightly shorter than corniculi, gradually tapering to tip, with lateral margins roughly fimbriated basally. Chelicera medium-sized, length of middle article 132–156; movable digit moderate sized (50–56, *lCh*/*lD* 8.65–9.71), slightly longer than corniculus (*lCh*/*lCo* 1.03–1.17). Fixed digit of chela ending in apical trident, masticatory surface with a row of 5–6 teeth in paraxial position and pilus dentilis in antaxial position (Figs 2–4). Movable digit bidentate. Palp length 180–204; internal seta of trochanter (39–43) slightly longer than external seta (34–38); palpi with typically specialized setae on femur (*al*) and genu (*al*1, *al*2) large, thick, with oblique tip.

*Legs*. Legs of moderate length (I 512–624, II 432–480, III 352–424, IV 512–592); leg I similar in length to dorsal shield, *lExI*/*lD* 0.91–1.09. Length of tarsi I 152–180, II 108–134, III 96–132, IV 160–192; tarsi of normal proportions: *lT*IV/*lD* 0.28–0.34, *lT*III/*wT*III 3.25–4.10. Leg II significantly thicker than others (width of genu II 56–68, genu I 36–40, genua III, IV 44–48). Leg chaetome in general as described for genus (Lindquist and Evans 1965); tibia IV with 7 setae, *pl* present. Most leg setae simple, of moderate lengths; length of *pd*2 on genu II (42–48) by a third shorter than width of segment (55–64). Tarsi II–IV each with some rather long setae (up to 100 on tarsus IV), many of them with fine tips (Figs 12, 14, 16); with lateral subapical setae *al*1 and *pl*1 needle-shaped, strong, rather long (24–32 on leg II, 21–39 on leg III, 36–56 on leg IV), two times more distant from tarsus apex and much thinner than robust blunt ventral subapical setae *av*1 and *pv*1 (14–19, 12–20, 16–27 respectively) (Figs 11–16); *av*2 on tarsus II and *pv*2 on tarsus III also thick, sometimes shortened, with blunt tips (Figs 11, 13). Ambulacrum I on pedicellate base, claws I (12–13) smaller than claws II-IV (16–21). Tarsus I distally with 6 rod-like solenidia, 4 of them inserted apically; length of sensillum with lanceolate apex 36–42. Ambulacra of legs II-IV (length 30–40) with rather long paradactyli (14–20 on legs II and III; 18–26 on leg IV) extending clearly beyond apices of claws. Tarsi II-IV with apical setae *ad*1 and *pd*1 (24–39 on legs II, III; 30–40 on leg IV) subequally as long as ambulacrum.

***Male*.** *Idiosomal dorsum*. Dorsal shield 446–552 × 228–260, more narrowed posteriad than in female (*lD*/*wD* ca 2.05–2.17); lateral incisions on average shorter than in female (20-40); chaetome in general as in female, but setae commonly slightly shorter (by 5–15 %); 9 pairs of marginal setae (*r*2-*r*5, *R*1-*R*5) on soft cuticle; *s*2 usually on soft cuticle, rarely on shield margin or absent.

*Idiosomal venter*. Tritosternum base (16–20 × 12–16) slightly smaller than in female (Fig. 17). Presternal platelets connected with sternitigenital shield. Sternitigenital shield united with endopodal platelets developed between coxae II-III, partly separated from ones situated between coxae III-IV and only abut ones developed between coxae I-II; its posterior margin slightly convex or truncate; length of shield 212–248, width 124–140, its narrowest part (44–48) between coxae IV. Sternitigenital shield without reticulation in the central and posterior areas, with setae *st*1–3 (30–38) longer than setae *st*4, 5 (20–28) and distinct lyrifissures *iv*1–3; vestiges of glands *gv*1 not discernible. Ventrianal shield well separated from sternitigenital and peritrematal shields, subtriangular, often with small anterior ledge, fully reticulated, its length 188–224, width 184–240, *lVa*/*wVa* 0.83–1.09, normally consolidated with metapodal platelet sigillae laterally; its anterolateral margins evenly convex, cribrum formed as in female. Ventrianal shield with 6 pairs of opisthogastral setae (*JV*1–4, *ZV*1, 2), 2–3 pairs of poroids and gland opening *gv*3, sometimes *ZV*1 asymmetrically absent, *JV*4 rarely asymmetrically off shield margin; *ZV*1, 2 (20–28) somewhat shorter than others (26–35), paranals 32–42, postanal seta 40–52; seta *JV*5 (34–40) on soft cuticle.

**Figures 5–10. F2:**
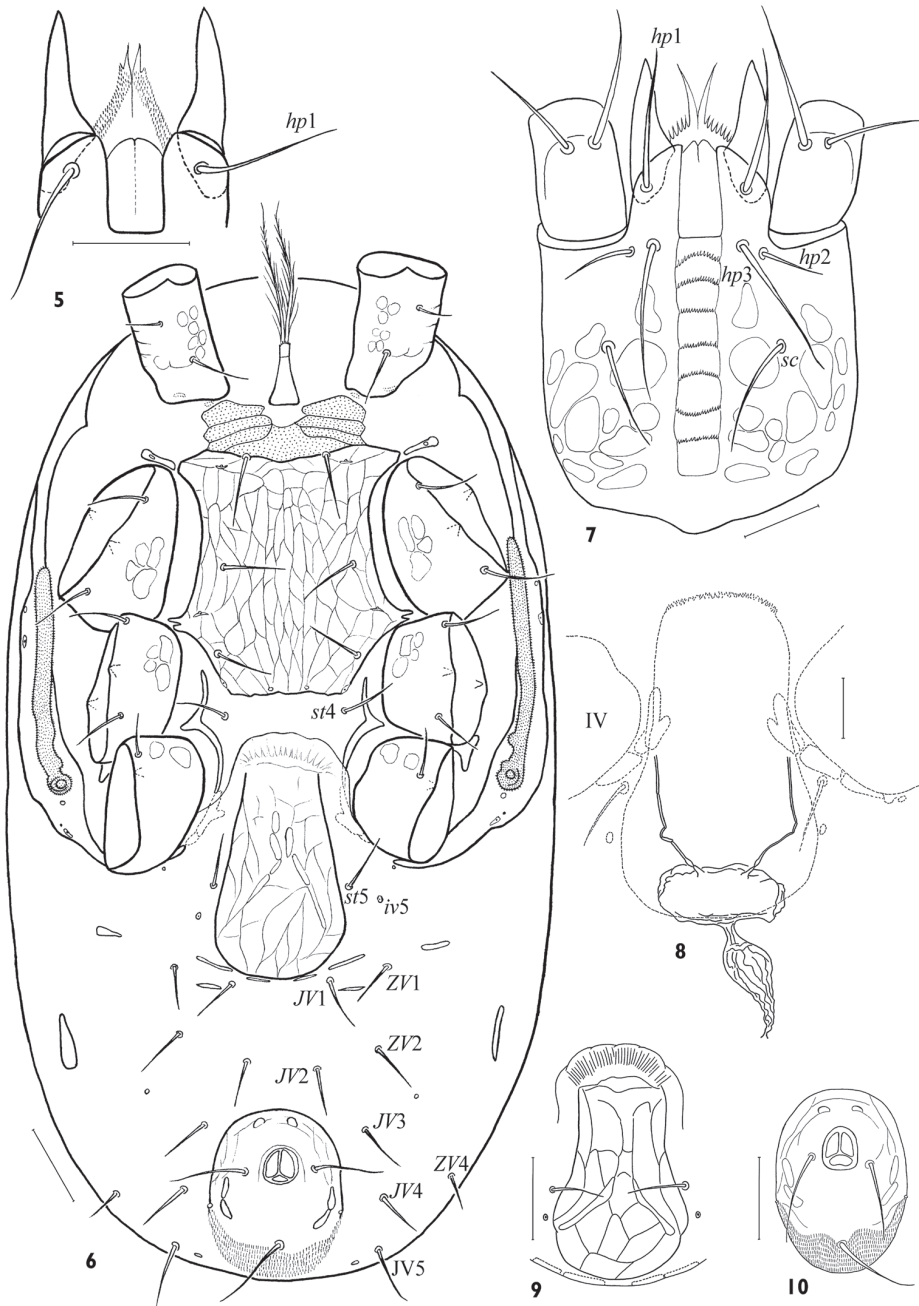
*Arctoseius koltschaki* sp. n., male **(5)** and female **(6–10) 5** hypostome **6** idiosomal venter **7** subcapitulum **8** inner part of spermathecal apparatus **9** variant of genital shield form **10** variant of anal shield form. Scales: **5, 7, 8** – 25 μm, **6, 9, 10** – 50 μm.

*Gnathosoma*. Gnathotectum as in female. Corniculi slightly thinner and more parallel than in female (Fig. 5). Internal malae unlike female finely and densely fimbriate. Cheliceral digits 46–52 (*lCh*/*lD* 8.58–10.94), longer than corniculus (42–47 × 12–15, *lCh*/*lCo* 1.02–1.30, *lCo*/*lD* 7.52–9.40). Fixed digit with dentition similar to female, but apical trident not developed and one denticle present subapically in antaxial position, apart from paraxial row of 5–6 denticles. Movable digit with one large denticle and tightly (at a sharp angle) sinuate spermatodactyl (length of free part 26–29), half protruding beyond tip of digit (Fig. 18).

*Legs*. Length of legs I to IV 492–596 (*lExI*/*lD* 0.96–1.13), 364–432, 336–416, and 472–568 respectively; length of tarsi I to IV 140–168, 96–124, 104–122, and 140–170 respectively. Leg II stouter than other legs, with dimorphically modified setae. Three setae on leg II, namely *v*2 on femur, *av*2 and *mv* on telotarsus, remarkably stout basally and attenuate apically, on elevated bases; opposable ventral setae *v*-1 on femur II and *av* on genu II and tibia II also usually slightly spinelike (Fig. 22). Other leg structure and setation generally as in female.

**Figures 11–16. F3:**
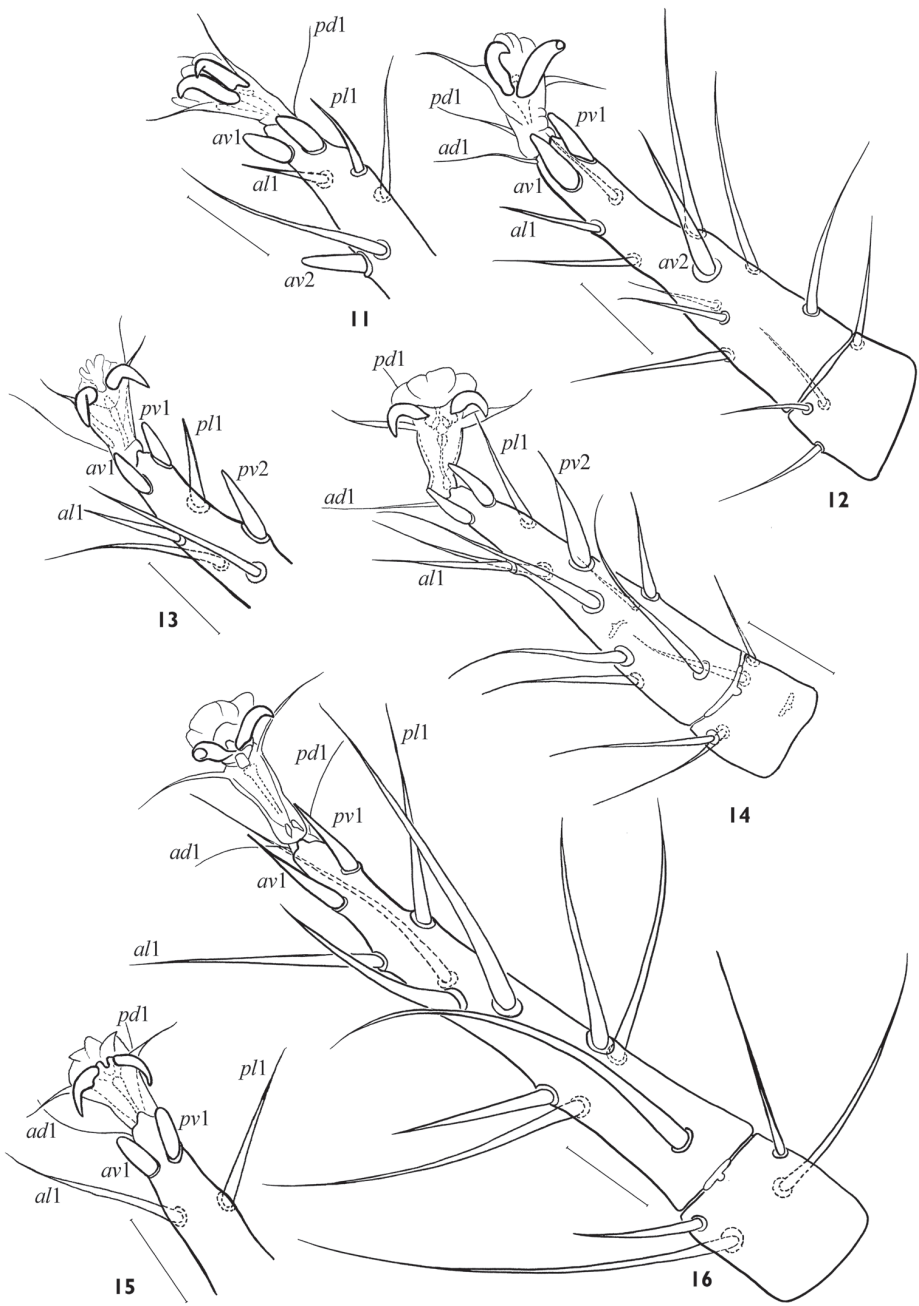
*Arctoseius koltschaki* sp. n., female **11, 12** variants of subapical setation of tarsus II **13, 14** variants of subapical setation of tarsus III **15, 16** variants of subapical setation of tarsus IV. Scales 25 μm.

**Figures 17–19. F4:**
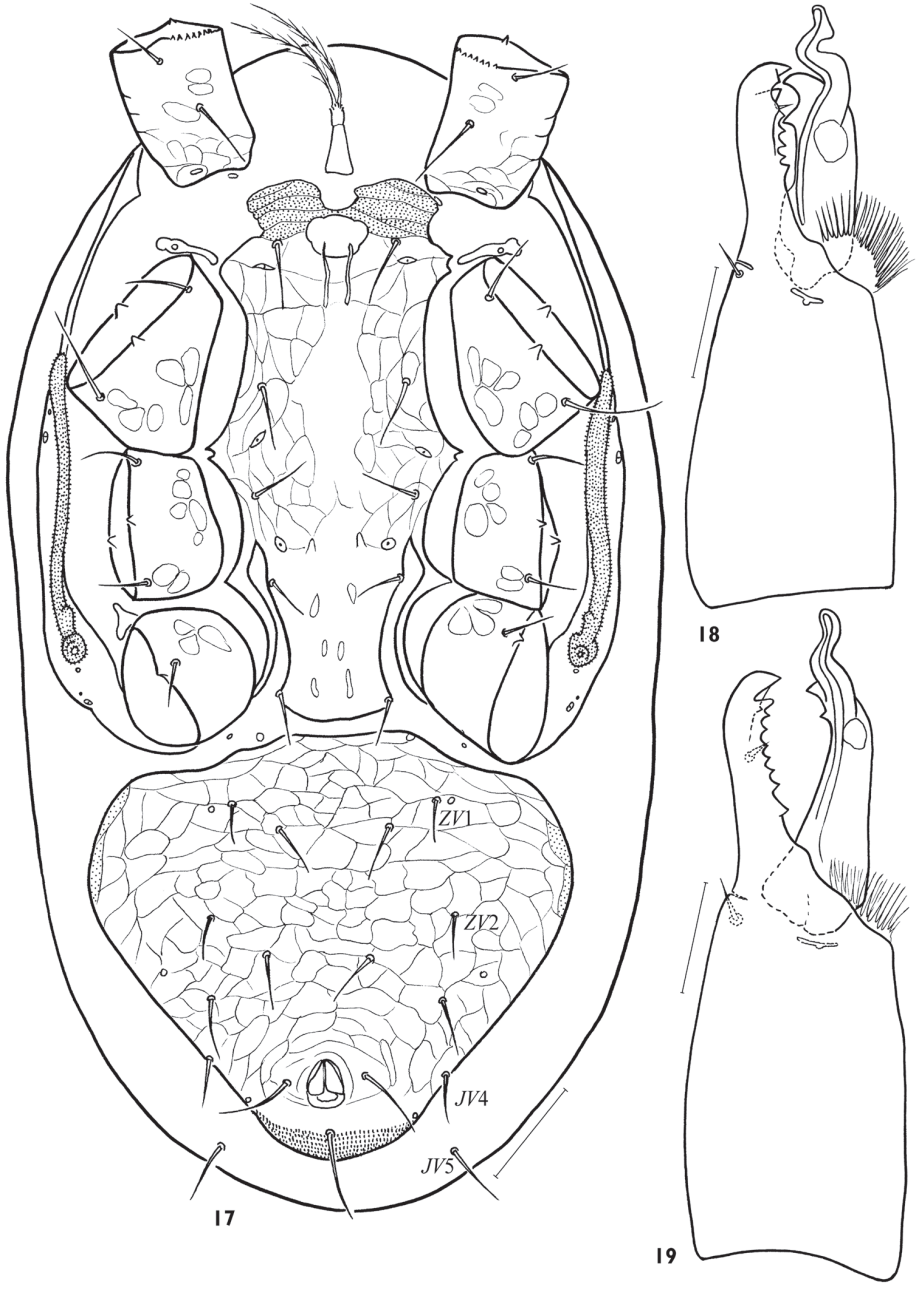
Males, *Arctoseius koltschaki* sp. n. **(17, 18)** and *Arctoseius multidentatus* Evans, 1955 **(19) 17** idiosomal venter **18, 19** chelicera. Scales: **17** – 50 μm, **18, 19** – 25 μm.

**Figures 20–24. F5:**
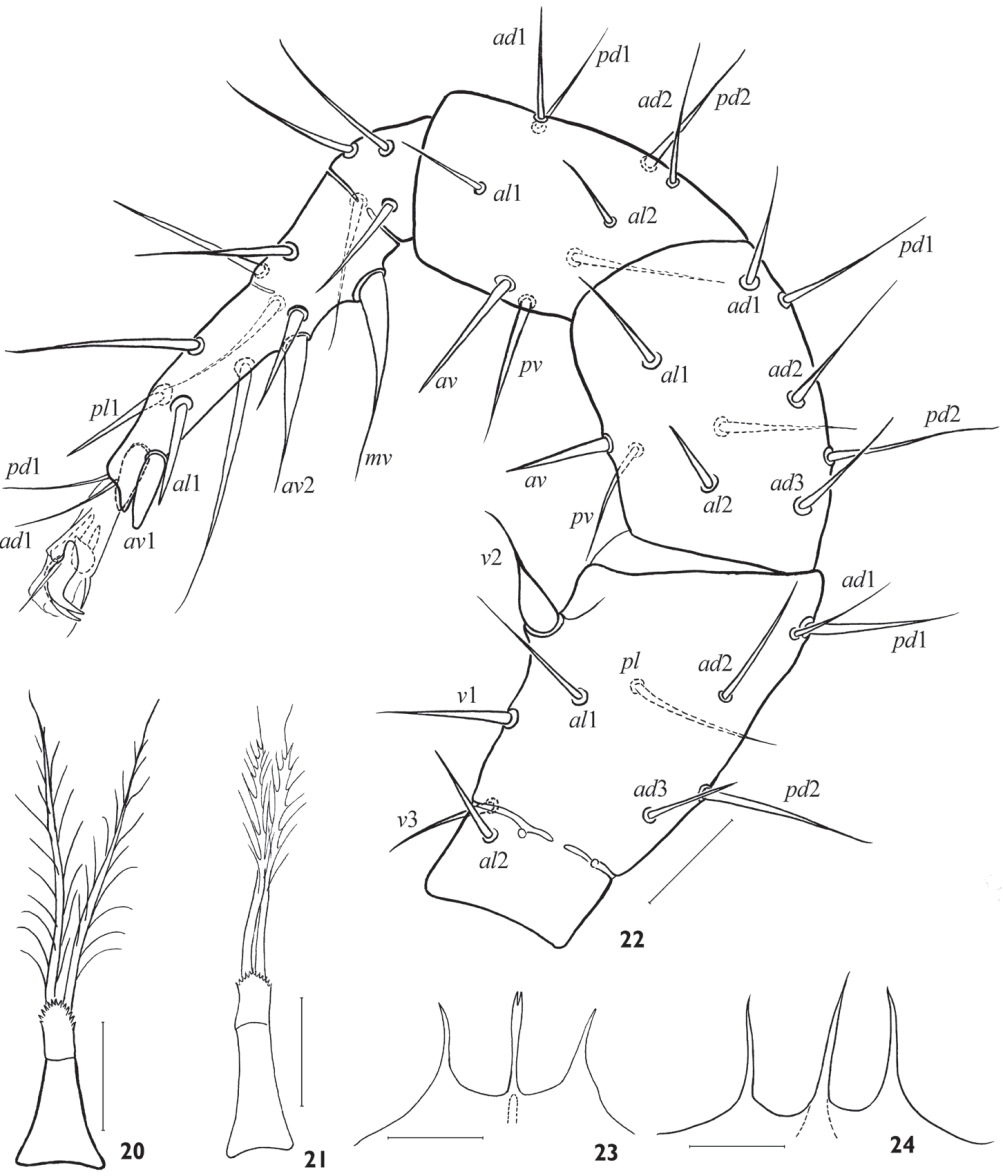
*Arctoseius koltschaki* sp. n., female **(20, 21, 23, 24)** and male **(22) 20, 21** tritosternum **22** leg II **23, 24** gnathotectum. Scales 25 μm.

**Figure 25.**
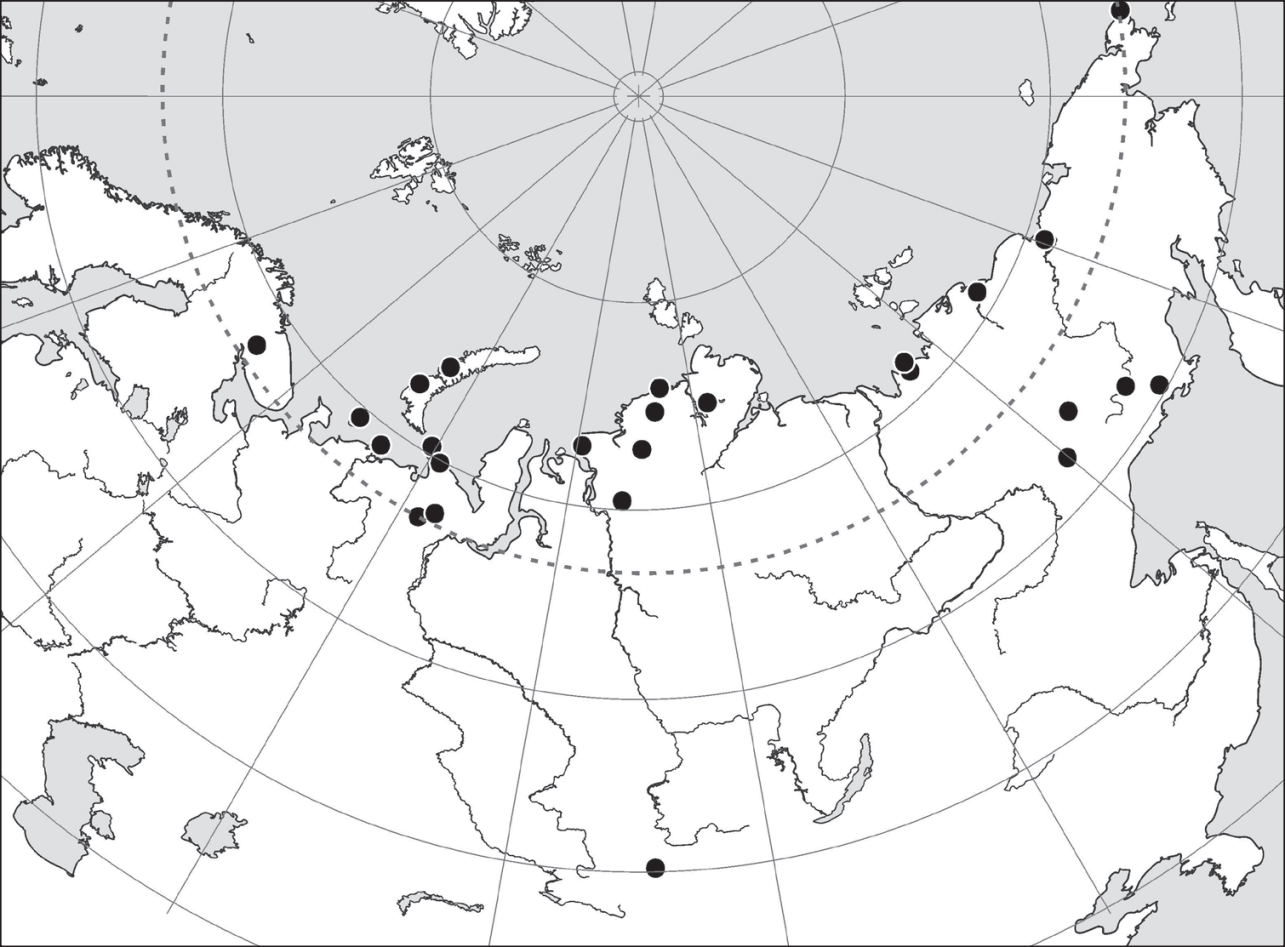
Records of *Arctoseius koltschaki* sp. n.

#### Differential diagnosis.

Adults of *Arctoseius koltschaki* sp. n. are similar to those of *Arctoseius multidentatus* Evans, 1955, but the dorsal shield in *Arctoseius koltschaki* sp. n. is often narrower (*lD*/*wD* in female 1.96–2.24, in male 2.05–2.17 instead of 1.91–2.05 and 1.85–2.03 respectively), and tarsi II-IV have subapical setae *av*1 and *pv*1 more stout and blunt (Figs 11–15). In male of *Arctoseius koltschaki* sp. n., the ventrianal shield is relatively wide (Fig. 17), subtriangular (narrowly oval in *Arctoseius multidentatus*), incorporating the metapodal platelets (free in *Arctoseius multidentatus*); the spermatodactyl is more tightly or sharply sinuate, and more angled apically (not as gently sinuate, and digitiform apically as in *Arctoseius multidentatus*) (cf. Figs 18 and 19). In female of *Arctoseius koltschaki* sp. n., the sternal shield is uniformly reticulated (whereas in *Arctoseius multidentatus*, the reticulation is developed laterally, and separately within an elongate posteromedial “window”). Female of *Arctoseius koltschaki* sp. n. is often smaller (dorsal shield length 528–616 instead of 566–688 in *Arctoseius multidentatus*).

#### Etymology.

The species named in honor of Admiral Alexander Koltschak, the prominent Arctic hydrologist and outstanding Citizen of Russia.

#### Distribution.

At present known from the Russian Arctic and Siberian mountain regions (Fig. 25).

#### Ecology.

Recorded from dry and humid tundra sites, various meadows including zoogenic ones (e.g. polar fox and lemming hills), snow-beds, bogs, larch forests and shrub (*Salix* spp., *Rhododendron aureum*, *Pinus pumila*) thickets.

#### Variability.

The species is rather stable morphologically. The form of female epigynal and anal shields varies a little (Figs 6, 9, 10). The variability concerns mainly the form of tectum (Figs 23, 24), the position of setae *s*2, *s*6, *S*3, *S*5 (on or off shield margin), and the form and size of leg subapical setae (Figs 11–16). The robust subapical setae *av*1 and *pv*1 sometimes may be acuminate, more often on leg IV (Fig. 16). The deutosternum rarely has 8 (instead normal 7) rows of denticles. The lacinia of trito-sternum seldom has adnate barbs (Fig. 21). One male (from 23 ones) originating from Chukotka has free baculiform metapodal platelets (26 × 4).

## Discussion. The *multidentatus*-group

### History

Previously and provisionally, there were some indications about the presence of new species close to *Arctoseius multidentatus* Evans, 1955, namely *Arctoseius* sp. aff. *multidentatus* [*Arctoseius koltschaki* sp. n.], «*Arctoseius haarlovi* Lindquist, 1963 ms.» (Lindquist 1964) [*Arctoseius haarlovi* Lindquist & Makarova, 2011], together forming a natural group. Their distinguishing features were given in a key to high-arctic *Arctoseius* species (Makarova 2000b). Recently Lindquist and Makarova (2011) augmented this group by the inclusion of *Arctoseius wisniewskii* Gwiazdowicz & Kamczyc, 2009 and *Arctoseius sexsetus* Lindquist & Makarova, 2011. Moreover, they pointed out the similarity between all mentioned species and *Arctoseius laterincisus* Thor, 1930, the type-species of the genus *Arctoseius*. In spite of its doubtful validity, this species can be formally considered as a member of the *multidentatus*-group, in view of its having the following features: shortened peritremes, elongated corniculi, and elongated posterior idiosomatic setae (Lindquist and Makarova 2011). However, subsequent to its original description, this species has not been found either on Svalbard (Ávila-Jiménez et al. 2011) or anywhere in the European Arctic (Makarova 2013). Very probably, its type series included specimens belonging to more than one species of *Arctoseius* (Lindquist and Makarova 2011). Because of these aspects, *Arctoseius laterincisus* is not included in the key below.

### Diagnosis

Idiosoma of moderate size (388–688 in females, 348–552 in males); dorsal shield rather narrow (*lD*/*wD* ca 1.9–2.6), not covering entire idiosoma, smooth, with distinct sigillae. Presternal platelets weakly sclerotized, lineate and punctate, fused with sternal shield. Sternal/sternitigenital shield often free from endopodal strips between coxae I–II (excluding *Arctoseius sexsetus*, *Arctoseius wisniewskii*), but united with them between coxae II–III. In females, anal shields small, usually not shorter than wide (*lAn*/*wAn* 0.85–1.40). In males, ventrianal shields with convex anterior margin, oval or roundish, leaving the metapodal platelets free on soft cuticle (exception – *Arctoseius koltschaki* sp. n. with wider, subtriangular ventrianal shield incorporating metapodal platelets). In both sexes, circumanal setae of similar lengths, with paranal setae relatively long, at least 0.7 as long as postanal seta. In both sexes, peritrematal shield reduced, of uneven width, with angulate extension between coxae I-II; peritreme shortened, extending anteriorly at most to mid-level of coxa II. Gnathotectum with three projections, these sometimes bifurcate apically. Corniculi rather long (*lCo*/*lD* ca 7–13), shorter or longer than cheliceral digits (*lCh*/*lCo* 0.74–1.44). In male, spermatodactyl straight or sinuate; leg II sometimes with dimorphically modified setae. Legs moderately long, leg I similar in length with dorsal shield.

The relatively long paranal setae are considered to be apomorphic for this species-group within the genus *Arctoseius*. The reduced peritremes and peritrematal shields, somewhat elongated corniculi, and complete lack of dorsal shield ornamentation (retention of deutonymphal condition) are also apomorphic, but are derived independently among some other *Arctoseius* species.

### Key to species of *Arctoseius* of *multidentatus*-group. Adults

**Table:** 

1	Podonotal seta *z*3 and opisthonotal seta *S*1 absent; corniculi large (*lCo*/*lD* 11.52–13.41), longer than cheliceral digits (*lCh*/*lCo* 0.74–0.93); fixed cheliceral digit with at most 4 denticles besides terminal hook; tibia IV with 6 setae, *pl* absent; in male, dimorphically modified setae on leg II absent, spermatodactyl straight	2
–	Podonotal seta *z*3 and opisthonotal seta *S*1 present (Fig. 1); corniculi smaller (*lCo*/*lD* 6.90–10.98), shorter than cheliceral digits (*lCh*/*lCo* 1.02–1.44); fixed cheliceral digit polydont, with 6–14 denticles besides terminal hook (Figs 4, 18); tibia IV with 7 setae, *pl* present; in male, dimorphically modified setae on leg II well developed (Fig. 22), spermatodactyl clearly sinuate (Figs 18, 19)	3
2	Fixed cheliceral digit terminating with trident, behind which one denticle present; in male, single denticle of movable digit situated in subapical position. Female 444–552 μm, male 404–464 μm. Alaska, Eurasian Arctic, Siberian mountains	*Arctoseius sexsetus* Lindquist & Makarova, 2011
–	Tip of fixed cheliceral digit of usual form, with three denticles forming a row behind apical hook; in male, single denticle of movable digit situated at a greater distance from apex, almost opposite pilus dentilis of fixed digit. Female 550 μm, male 500 μm, Stolovye Mountains, Poland	*Arctoseius wisniewskii* Gwiazdowicz & Kamczyc, 2009
3	Smaller mites, dorsal shield length at most in female 465 μm, in male 420 μm; opisthonotal region with 13 pairs of setae (only one pair of setae present in area usually occupied by *J*4 and *Z*4); peritreme much reduced, usually not extending beyond mid-level of coxa III anteriorly. Female 388–465 μm, male 348–420 μm. Circumpolar	*Arctoseius haarlovi* Lindquist & Makarova, 2011
–	Larger mites, dorsal shield length at least in female 528 μm, in male 446 μm; opisthonotal region with 14 pairs of setae (*J*1–5, *Z*1–5, *S*1, 3–5); peritreme terminating about mid-level of coxa II anteriorly (Figs 6, 17)	4
4	Tarsi II-IV with subapical setae *av*-1 and *pv*-1 stout and blunt (Figs 11–15); in male, ventrianal shield relatively wide, subtriangular, incorporating metapodal platelets (Fig. 17); spermatodactyl tightly sinuated, and angled apically (Fig. 18). Female 528–616 μm, male 446–552 μm. Eurasian Arctic and Metaarctic, South Siberia (West Sayan Mountains)	*Arctoseius koltschaki* sp. n.
–	Tarsi II-IV with subapical setae *av*-1 and *pv*-1 needle-shaped, but thicker than *pl*-1, *al*-1; in male, ventrianal shield usually narrow, often oval, leaving metapodal platelets free on soft cuticle; spermatodactyl more gently sinuated, and either simple or bluntly angled apically (Fig. 19). Female 566–688 μm, male 488–550 μm. Circumpolar	*Arctoseius multidentatus* Evans, 1955

## Supplementary Material

XML Treatment for Arctoseius koltschaki
